# Almonertinib plus chemotherapy *versus* almonertinib alone in second-line treatment of advanced non-small cell lung cancer with mutated epidermal growth factor receptor: a retrospective study

**DOI:** 10.3389/fonc.2023.1248690

**Published:** 2023-09-11

**Authors:** Xiaoxu Fang, Yan Xiang, Kaihua Lu

**Affiliations:** Department of Oncology, The First Affiliated Hospital of Nanjing Medical University, Nanjing, China

**Keywords:** almonertinib, non-small cell lung cancer, epidermal growth factor receptor mutation, chemotherapy, safety

## Abstract

**Objective:**

This study mainly observes the efficacy and safety of almonertinib plus chemotherapy compared with almonertinib alone in the second-line treatment of advanced non-small cell lung cancer (NSCLC) with mutated epidermal growth factor receptor (EGFR).

**Methods:**

In this study, clinical data of 68 patients with advanced NSCLC who were treated in Jiangsu Provincial People’s Hospital and Nanjing Chest Hospital between April 2020 and December 2022 were collected. Among them, the study group (n=30) received second-line almonertinib combined with platinum-based chemotherapy, while the control group (n=38) received almonertinib alone. The near-term and long-term effects and adverse events of the two groups were compared respectively.

**Results:**

The median follow-up time until 31 December 2022 was 16.3 months (95% CI: 11.32-21.34). Results of chi-square analysis showed no statistically significant difference in objective response rate (ORR) and disease control rate (DCR) between the study group and the control group (56.73% *vs.* 55.3%, *P>0.05*; 100% *vs.* 86.8%, *P>0.05*). Log-rank test comparing the two groups revealed that the median progression-free survival (mPFS) of the study group was significantly longer than that of the control group by 3.1 months (12.7 *vs.* 9.6 months, *P=0.01*). Multivariate COX proportional risk model showed a statistically significant effect of treatment method and PS score on PFS (HR=0.43, *P=0.023*; HR=3.82, *P=0.001*). In terms of safety, most of the adverse events (AEs) were mild, with no grade 4-5 in the two groups, and the overall tolerance of patients was good.

**Conclusion:**

For advanced NSCLC patients with EGFR mutations, second-line treatment with almonertinib plus chemotherapy significantly improved PFS compared with almonertinib alone without a significant increase in adverse events, providing efficacy and safety.

## 1 Introduction

Lung cancer has the second highest incidence rate (11.4%) and the highest mortality rate (18%) among all cancers, of which non-small cell lung cancer (NSCLC) accounts for about 85% (1). In recent years, the development of genetic testing technology has driven research at the molecular biology level of lung cancer, and targeted therapy for advanced NSCLC has thus entered a new era. Epidermal growth factor receptor (EGFR) is an important driver gene of NSCLC and plays an important role in its development. Numerous clinical studies have confirmed that compared with traditional platinum-based chemotherapy, EGFR tyrosine kinase inhibitors (EGFR-TKIs) exhibit significant advantages on objective response rate (ORR) and progression free survival (PFS) in the treatment of advanced NSCLC patients with EGFR mutations (2–5). However, most patients develop resistance to EGFR-TKIs after 9 to 13 months (6). Current studies have found that the mechanisms of resistance are complex and varied, such as T790M mutation, MET amplification, HER2 amplification, activation of downstream signaling pathways and changes in histological types, etc. (7).

The third-generation EGFR-TKIs can bind to cysteine-797 (Cys-797) and are highly selective for EGFR-sensitive mutations and EGFR T790M-resistant mutations (8). As a third-generation EGFR-TKI independently developed in China, the structure of almonertinib is further optimised compared with osimertinib by replacing the methyl group with a cyclopropyl group on the indole nitrogen ring, so that it does not inhibit the wild-type EGFR (WT-EGFR) during metabolism, and metabolite production is reduced. In terms of adverse events (AE), almonertinib has a better overall safety (9–11).

Based on the APOLLO study, almonertinib was approved in China in March 2020 for second-line treatment of advanced or metastatic NSCLC patients with EGFR T790M mutation (12). Subsequent results from the AENEAS study showed that almonertinib also had great efficacy in the first-line treatment of advanced or metastatic NSCLC patients with EGFR exon 19 deletion or exon 21 (L858R) mutation (13). However, there are few real-world studies on the efficacy and safety of almonertinib. In this study, 68 patients with advanced NSCLC with EGFR mutations diagnosed and treated at Jiangsu Provincial People’s Hospital and Nanjing Chest Hospital from April 2020 to December 2022 were collected as study subjects to compare the efficacy and safety of almonertinib plus chemotherapy and almonertinib alone, to provide reference for second-line treatment of advanced NSCLC patients. The results are reported as follows.

## 2 Methods

### 2.1 Patient selection

Patients who were diagnosed with advanced or metastatic NSCLC (stage IIIB/IV) and had undergone molecular testing at Jiangsu Provincial People’s Hospital or Nanjing Chest Hospital from April 2020 to December 2022 were reviewed in our retrospective study. EGFR mutation was detected through the method of amplification refractory mutation system-polymerase chain reaction (ARMS-PCR) by Multi-Gene Mutations Detection Kit (AmoyDx, Xiamen, China) or through next-generation sequencing (NGS) via Illumia Hiseq platform (Geneseeq, Nanjing, China). All patients had failed (or progressed) on first-line treatment and had at least one target lesion that can objectively evaluate the efficacy.

### 2.2 Treatment method

Patients in the study group received second-line almonertinib (110 mg once daily) combined with platinum-based chemotherapy for 21 days as a cycle, and patients in the control group received only almonertinib (110 mg once daily). Patients should complete baseline examinations at the time of definitive diagnosis, including tumor markers, enhanced computed tomography (CT) of the chest and abdomen, magnetic resonance imaging (MRI) of the head, and bone emission CT(ECT) or positron emission CT(PET-CT), and conduct comprehensive evaluations every two months thereafter. Patients were observed for treatment efficacy and related adverse events until tumor progression or intolerable adverse events occurred.

### 2.3 Response assessment

Solid tumor efficacy evaluation criteria (RECIST 1.1) were used to evaluate the efficacy of patients’ lesions, which were classified into complete response (CR), partial response (PR), stable disease (SD) and progressive disease (PD). Objective response rate (ORR) = CR + PR and disease control rate (DCR) = CR + PR + SD. progression-free survival (PFS) was defined as the time between the start of almonertinib combination chemotherapy or monotherapy and the time of disease progression or patient death. Patients were followed up by telephone to record adverse events during treatment and graded for adverse events according to the National Cancer Institute Toxicity Classification Criteria (NCI-CTC) 5.0. The last follow-up time was in December 2022.

### 2.4 Statistical analysis

Normally distributed quantitative information was expressed as mean ± standard deviation (Mean ± SD) and independent samples t-tests were used to compare differences between groups. Qualitative information was compared between the two rates using the chi-square test. The Kaplan-Meier method was used to plot survival curves, and the Log-rank test was used to compare survival between groups. The COX proportional risk model was used to look for independent risk factors affecting the prognosis. *P<0.05* was considered statistically significant.

### 2.5 Ethics

This study was a retrospective study reviewed and approved by the ethics committee of the First Affiliated Hospital of Nanjing Medical University. Informed consent was obtained from all individual participants included in the study.

## 3 Results

### 3.1 Clinical and molecular characteristics

A total of 191 cases of NSCLC patients treated with almonertinib at Jiangsu Provincial People’s Hospital and Nanjing Chest Hospital from April 2020 to December 2022 were collected, and a total of 68 cases were finally included in the analysis based on the criteria, including 38 cases in the control group and 30 cases in the study group. A greater proportion of these patients were women (n=43, 63.2%) and never-smokers (n=61, 89.7%), with a median age of 63 years (range: 51–75years). 8 cases (11.8%) in stage IIIB and 60 cases (88.2%) in stage IV. There were 35 cases (51.5%) with EGFR exon 19 deletion (19del), 28 cases (41.2%) with EGFR exon 21 mutation and 5 cases (7.4%) with uncommon mutation. T790M mutation was found in 32 (47.1%) patients. 29 (42.6%) patients had central nervous system (CNS) metastases. Extrapulmonary metastases to ≤2 organs were present in 63 patients (89.7%).PS scores of 0-1 were present in 42 patients (61.8%) and ≥2 in 26 patients (38.2%). Previous treatment with first generation of EGFR-TKIs was received by 52 patients (76.5%). The baseline characteristics of the 68 patients are shown in **Table 1**. There was no statistically significant difference between the control group and the study group in terms of gender, age, smoking history, histopathological type, TMN stage, gene mutation, PS score, previous treatment, CNS metastasis, and extrapulmonary metastasis (*P>0.05*), and the two groups were well balanced.

**Table 1:** Clinical and molecular characteristics.

Characteristics	Total	Control group	Study group	*P*	*t/χ^2^*
Total	68	38	30	1.000	
Gender				0.988	<0.05
Male	25 (36.8%)	14 (36.8%)	11 (36.7%)		
Female	43 (63.2%)	24 (63.2%)	19 (63.3%)		
Age	63 ± 12	66 ± 13	59 ± 9	0.17	0.974
Smoking history				0.053	3.757
No	61 (89.7%)	37 (97.4%)	24 (80.0%)		
Yes	7 (10.3%)	1 (2.6%)	6 (20.0%)		
Genetic mutation				0.112	4.38
Exon 19	35 (51.5%)	23 (60.5%)	12 (40.0%)		
Exon 21	28 (41.2%)	14 (36.8%)	14 (46.7%)		
Uncommon	5 (7.4%)	1 (2.6%)	4 (13.3%)		
T790M mutation				0.584	0.30
Yes	32 (47.1%)	19 (50.0%)	13 (43.3%)		
No	36 (52.9%)	19 (50.0%)	17 (56.7%)		
CNS metastasis				0.695	0.154
Yes	29 (42.6%)	17 (44.7%)	12 (40.0%)		
No	39 (57.4%)	21 (55.3%)	18 (60.0%)		
Extrapulmonary metastasis				0. 509	0.436
≤2	63 (92.6%)	34 (89.5%)	29 (96.7%)		
>2	5 (7.4%)	4 (10.5%)	1 (3.3%)		
PS score				1.0	<0.05
0-1	61 (89.7%)	34 (89.5%)	27 (90.0%)		
≥2	7 (10.3%)	4 (10.5%)	3 (10.0%)		
TNM Stage				1.0	<0.05
III	8 (11.8%)	4 (10.5%)	4 (13.3%)		
IV	60 (88.2%)	34 (89.5%)	26 (86.7%)		
Previous treatment				0.236	1.405
First-generation TKIs	52 (76.5%)	27 (71.1%)	25 (83.3%)		
Others	16 (23.5%)	11 (28.9%)	5 (16.7%)		

### 3.2 Results

As of December 2022, the median follow-up time was 16.3 months (95% CI: 11.32-21.34). Of the 68 patients with stage IIIB/IV NSCLC, 38 patients were assessed for PR (55.9%), 25 for SD (36.8%), and 5 for PD (7.4%). The ORR was 55.9% (38/68) and DCR was 92.6% (63/68). The mPFS was 11.5 months (95% CI:8.77-14.23).

Results of subgroup analyses of near-term efficacy: 21 patients in the control group were assessed for PR (55.3%), 12 (31.6%) for SD, 5 (13.2%) for PD. The ORR was 55.3% (21/38) and DCR was 86.8% (33/38). 17 patients in the study group were assessed for PR (56.7%), 13 (43.3%) for SD, no patients assessed as PD. The ORR was 56.7% (17/30) and DCR was 100% (30/30). The chi-square test suggested that there was no statistically significant difference in ORR and DCR between the study group and control group (*χ^2^=0.013, P=0.908; χ^2^=2.548, P=0.11*) (**Table 2**).

**Table 2:** Comparison of near-term and long-term efficacy between two groups of patients.

Efficacy	Control group	Study group	*χ^2^*	*P*
CR	0	0		
PR	21 (55.3%)	17 (56.7%)		
SD	12 (31.6%)	13 (43.3%)		
PD	5 (13.2%)	0 (0.0%)		
ORR	21 (55.3%)	17 (56.7%)	0.013	0.908
DCR	33 (86.8%)	30 (100%)	2.548	0.11
mPFS(m)	9.6(95%CI: 7.92-11.28)	12.7(95%CI:11.34-14.00)		

**Figure 1:**
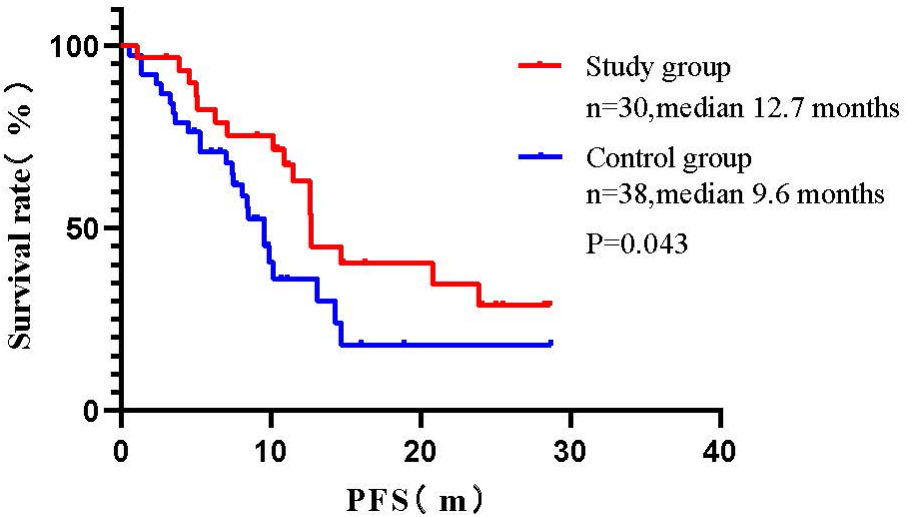
Comparison of survival curves between two groups of patients (Log-rank test).

Results of subgroup analysis of long-term efficacy: The mPFS of the control group patients was 9.6 months (95% CI: 7.92-11.28), while the mPFS of the study group was 12.7 months (95% CI: 11.34-14.00). Log-rank test compared the two groups found that: patients in the study group had a significant benefit in terms of PFS when compared with the control group (mPFS:12.7 *vs.* 9.6 months, *P=0.043*) (**Figure 1**).

### 3.3 Univariate and multivariate Cox regression analysis of PFS

**Figure 2:**
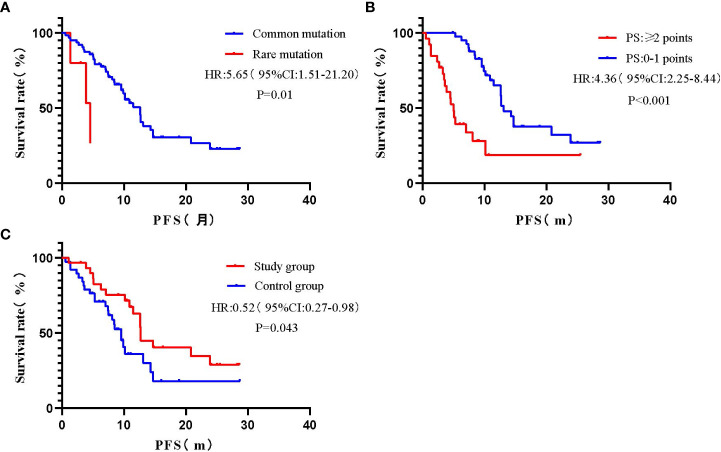
Survival curves for univariate analysis of different variables. **(A)** Gene mutation; **(B)** PS score; **(C)** Treatment method.

Univariate COX regression analysis showed that gender, smoking history, TNM stage, T790M mutation, CNS metastasis, and extrapulmonary metastasis had no statistically significant impact on PFS (*P>0.05*), while gene mutation, PS score, and treatment method had a statistically significant impact on PFS (*P<0.05*) (**Figure 2**, **Table 3**).

**Table 3:** Univariate and multivariate Cox regression analysis of PFS (N=68).

Variables	Group	Univariate analysis		Multivariate analysis	
		HR (95%CI)	*P* value	HR (95%CI)	*P* value
Gender	Male				
	Female	1.00 (0.53-1.91)	0.98		
Smoking history	N0				
	Yes	0.59 (0.18-1.92)	0.38		
TMN Stage	III				
	IV	2.29 (0.71-7.45)	0.17	2.22 (0.69-8.68)	0.168
Gene mutation	Common				
	Uncommon	5.65 (1.51-21.20)	0.01	4.35 (0.99-19.11)	0.051
T790M mutation	No				
	Yes	0.79 (0.43-1.46)	0.46	0.69 (0.35-1.37)	0.693
PS score	0-1				
	≥2	4.36 (2.25-8.44)	<0.001	3.82 (1.71-8.54)	0.001
CNS metastasis	No				
	Yes	1.27 (0.69-2.35)	0.45	0.92 (0.47-1.81)	0.806
Extrapulmonary metastasis	≤2				
	>2	1.73 (0.61-4.90)	0.31	0.76 (0.23-2.46)	0.644
Treatment method	Control group				
	Study group	0.52 (0.27-0.98)	0.043	0.43 (0.21-0.89)	0.023

A multivariate COX proportional risk model was constructed by incorporating variables such as TNM stage, T790M mutation, CNS metastasis, extrapulmonary metastasis, gene mutation, PS score, and treatment method (**Table 3**). The results found that the effect of uncommon mutations on PFS was not statistically significant compared to common mutations (HR=4.35, 95% CI: 0.99-19.11, *P=0.051*), which was inconsistent with the results of the univariate COX regression analysis. In addition, patients with PS ≥ 2 had a statistically significant higher risk of death compared to patients with PS 0-1 (HR=3.82, 95% CI: 1.71-8.54, *P=0.001*); Besides, patients in the study group had a statistically significantly longer PFS than those in the control group (HR= 0.43, 95% CI: 0.21-0.89, *P = 0.023*); The effects of other variables on PFS were not statistically significant.

### 3.4 Safety

Adverse events were mild in most of patients, predominantly grade 1-2, with a low incidence of grade 3 and no grade 4-5 observed (**Table 4**). Common adverse events of almonertinib include rash (22.1%), increased blood creatine kinase (CK) levels (17.6%), diarrhea (8.8%), nausea (8.8%), decreased appetite (7.4%) and oral ulcers (7.6%), etc. The combination of chemotherapy increases myelosuppression, gastrointestinal reactions, and hepatotoxicity. 68 patients did not experience discontinuation of the drug due to serious adverse events or treatment-related related deaths, thus showing the safety of the two methods in treating patients with advanced NSCLC with EGFR mutations.

**Table 4:** Adverse events during treatment in 68 patients.

Adverse events	Grade 1-2		Grade 3			
	Control group	Study group	Control group	Study group	Total (%)	
Oral ulcers	3	2	0	0	5 (7.6%)	
Rash	8	7	0	0	15 (22.1%)	
Diarrhea	1	4	1	0	6 (8.8%)	
Nausea	1	3	2	0	6 (8.8%)	
Myelosuppression	3	9	0	7	19 (27.9%)	
Hepatotoxicity	0	6	0	0	6 (8.8%)	
increased CK	5	7	0	0	12 (17.6%)	
Fatigue	1	2	0	0	3 (4.4%)	
Decreased appetite	1	4	0	0	5 (7.4%)	

## 4 Discussion

T790M mutation is the most common resistance mechanism of first- and second-generation EGFR-TKIs. As a third-generation EGFR-TKI that selectively acts on EGFR-sensitive mutations and secondary T790M resistant mutations, almonertinib has demonstrated excellent safety and efficacy in large-scale clinical trials. The APOLLO trial, as the first phase II clinical study to evaluate the efficacy of almonertinib in the second-line treatment, found that the ORR of almonertinib in NSCLC with EGFR T790M resistant mutation was 68.9% (95% CI: 62.6-74.6), and the DCR was 93.4% (95% CI: 89.6-96.2). The median druation of response (mDOR) was 15.1 months (95% CI: 12.5-16.6) and mPFS was 12.4 months (95% CI: 9.7-15.0) (12).The AENEAS study found that almonertinib significantly prolonged PFS compared with gefitinib (mPFS: 19.3 *vs.* 9.9 months, HR=0.46, *P<0.0001*), and there was no significant difference in ORR (73.8% *vs.* 72.1%) and DCR (93.0% *vs.* 96.7%) between the two groups. The above two studies demonstrated that almonertinib significantly improved PFS and DOR in patients with EGFR-mutated advanced NSCLC both as a first- and second-line treatment, and was well tolerated by patients (13).

However, the efficacy of almonertinib in clinical practice has not been widely reported. In this study, we retrospectively analysed the efficacy of 68 cases of EGFR-positive advanced NSCLC treated with almonertinib in the second line. The results showed that the ORR was 55.9%, DCR was 92.6%, and the mPFS was 11.5 months (95% CI: 8.77-14.23). Comparing the results of the APOLLO study, it was evident that the efficacy of almonertinib in clinical practice could not reach the desired effect of the study. We considered that it might be related to the baseline conditions of the enrolled patients. The PS scores of the enrolment conditions of the APOLLO study were all 0-1, while 38.2% of the patients enrolled in this study had PS scores of ≥2. The COX multifactorial analysis suggested that patients with a PS score of 0-1 had an elevated risk of death compared with those with a PS ≥2, and the difference was statistically significant (HR= 3.82, 95% CI: 1.71-8.54, *P = 0.001*).

Five patients with uncommon mutations were included in this study, including EFGR 18exon G719X, 21exon L833V/H835L, 21exon L861Q, and 20exon insertion mutation. Previous studies concluded that patients with common mutations usually have better treatment response and longer PFS on EGFR-TKIs compared to patients carrying uncommon mutations (14). In this study, we concluded by Log-rank test that the mPFS of patients with uncommon mutations was 4.6 months, while that of patients with common mutations was 12.57 months (95% CI: 9.87-15.27 months), and the difference was statistically significant (*P=0.022*). However, the results of COX multifactorial analysis showed that EGFR uncommon mutations did not have a statistically significant effect on PFS compared to EGFR common mutations (HR=4.35, 95% CI: 0.99-19.11, *P=0.051*). It should be noted that there are few preclinical data on the efficacy of almonertinib in the treatment of uncommon EGFR mutations, and due to the small number of observed cases in this study, information on the mutations in some of the patients was not available. Therefore, the efficacy of almonertinib in treating patients with uncommon mutations still needs to be further verified by expanding the sample size at a later stage.

To our knowledge, this study is the first to explore the efficacy and safety of almonertinib combined with platinum-based chemotherapy in the treatment of advanced NSCLC with EGFR mutations compared to almonertinib monotherapy. As a second-line treatment, there was no statistically significant difference in ORR and DCR as far as near-term efficacy was concerned, and in terms of long-term efficacy, the PFS was significantly prolonged by almonertinib combination chemotherapy versus almonertinib alone (mPFS: 12.67 *vs.* 9.6 months, *P=0.046*). Therefore, the regimen of almonertinib in combination with chemotherapy may further improve the prognosis of patients. However, due to the lack of OS-corresponding data and small sample size in this study, the efficacy of almonertinib still needs to be further observed.

In terms of safety, adverse events of almonertinib were also associated with treatment-related rash (any grade, 22.1%) and diarrhea (any grade, 8.8%) compared with osimertinib in the AURA2 study (15). However, patients had a greater incidence of increased blood creatine kinase levels (any grade, 17.6%), and it is noteworthy that all of these patients had less than grade 3 adverse events, and no patients discontinued almonertinib due to this AE. The remaining common adverse events included diarrhea (8.8%), nausea (8.8%) decreased appetite (7.4%) and oral ulcers (7.6%), etc. which is more consistent with previous studies (13). The combination of chemotherapy increases myelosuppression, gastrointestinal reaction and hepatotoxicity. Most of the adverse events were mild, with no grade 4-5 in the whole group, and the overall tolerability was good. Due to the small number of patients enrolled, further observation is still needed to assess the risks associated with treatment.

## 5 Summary

This study still has the following shortcomings: Firstly, as a retrospective analysis, the clinical sample size of this study was small (n=68), and different individuals received different specific platinum-based chemotherapy therapies, which may affect the evaluation of the efficacy of the treatment; Secondly, the potential selection bias and the limitations of the statistical test also affect the comprehensive evaluation of the patients’ conditions to a certain extent; Thirdly, the gene mutation information of some patients were not known, and the impact on the patients’ prognosis could not be accurately evaluated, which has to be further verified by enlarging the sample size in a later period of time.

To sum up, for advanced NSCLC patients with EGFR mutations, second-line treatment with almonertinib plus chemotherapy significantly improved PFS compared with almonertinib alone without a significant increase in adverse events, providing efficacy and safety.

## Data availability statement

The original contributions presented in the study are included in the article/supplementary materials, further inquiries can be directed to the corresponding author.

## Ethics statement

This study was approved by the ethics committee of the First Affiliated Hospital of Nanjing Medical University. The studies were conducted in accordance with the local legislation and institutional requirements. Written informed consent for participation was not required from the participants or the participants’ legal guardians/next of kin in accordance with the national legislation and institutional requirements. Written informed consent was obtained from the individual(s) for the publication of any potentially identifiable images or data included in this article.

## Author contributions

Conceptualization: XF and YX; data curation: XF; writing—original draft preparation: YX; writing—review and editing: YX and KL; funding acquisition: KL. All authors contributed to the article and approved the submitted version.
